# Serovars, Genetic Relatedness and Antimicrobial Resistance of Non-Typhoidal *Salmonella* in Poultry and Farm Workers in Southeastern Nigeria

**DOI:** 10.3390/microorganisms14040850

**Published:** 2026-04-09

**Authors:** Ifeyinwa R. Okosi, Onyinye J. Okorie-Kanu, Lynda Majesty-Alukagberie, Chinazom M. Eze, Chidiebere Anyaoha, Obichukwu C. Nwobi, Onyinye Onwumere-Idolor, Temitope M. Ogunniran, George N. Anosa, Toyin Olubade-Olatokunbo, Onyemaechi Ugboh, Simeon C. Okafor, Obianuju Okoroafor, Nkechi H. Ikena-Ezeh, Uju C. Okafor, Madubuike U. Anyanwu, Charles Odilichukwu R. Okpala

**Affiliations:** 1 Department of Diagnostic and Outstation Services, National Veterinary Research Institute, P.O. Box 01, Vom 930101, Nigeria; ify.okosi@nvri.gov.ng (I.R.O.); toyin.olubade@nvri.gov.ng (T.O.-O.); 2 Department of Veterinary Public Health and Preventive Medicine, University of Nigeria, Nsukka 402001, Nigeria; lynda.majesty-alukagberie@unn.edu.ng (L.M.-A.); chidi.anyaoha@unn.edu.ng (C.A.); obichukwu.nwobi@unn.edu.ng (O.C.N.); okaforujucatherine@gmail.com (U.C.O.); 3 Department of Food Science and Technology, University of Nigeria, Nsukka 402001, Nigeria; chinazom.eze@unn.edu.ng; 4 Department of Animal Production, Faculty of Agriculture, Southern Delta University, PMB 005, Ozoro 501103, Nigeria; onwumere-idolors@dsust.edu.ng; 5 Department of Veterinary Medicine, University of Nigeria, Nsukka 402001, Nigeria; temitope.ogunniran@unn.edu.ng (T.M.O.); george.anosa@unn.edu.ng (G.N.A.); obianuju.okoroafor@unn.edu.ng (O.O.); 6 Department of Agricultural Economics and Extension, University of Delta, P.O. Box 2090, Agbor 321101, Nigeria; onyemaechi.ugboh@unidel.edu.ng; 7 Department of Veterinary Pathology, University of Nigeria, Nsukka 402001, Nigeria; simeon.okafor@unn.edu.ng; 8 Department of Veterinary Microbiology and Immunology, University of Nigeria, Nsukka 402001, Nigeria; nkechi.ikenna-ezeh@unn.edu.ng (N.H.I.-E.); madubuike.anyanwu@unn.edu.ng (M.U.A.); 9 UGA Cooperative Extension, College of Agricultural and Environmental Sciences, University of Georgia, Athens, GA 30602, USA

**Keywords:** antimicrobial resistance, ERIC-PCR, Nigeria, non-typhoidal *Salmonella*, One Health, poultry production

## Abstract

Non-typhoidal *Salmonella* (NTS) is an important poultry-associated pathogen with major One Health and economic impacts, but data on its epidemiology and antimicrobial resistance in Nigeria remain limited. This study investigated the prevalence, serovar distribution, clonal relatedness, and antimicrobial resistance of NTS along the poultry production chain in Enugu State, southeastern Nigeria. A total of 2400 samples were collected, comprising feces (cecal content)/cloacal swabs from chickens (*n* = 1100), eggs (*n* = 400), chicken meat (*n* = 600), and stool samples from poultry workers (*n* = 300). Isolation and identification were performed using standard bacteriological methods, with confirmation by serotyping and polymerase chain reaction (PCR) targeting the *invA* gene. Genetic relatedness was assessed using enterobacterial repetitive intergenic consensus (ERIC)-PCR, and antimicrobial susceptibility was determined by the disk diffusion method. Overall, 47 (2.0%) *Salmonella enterica* isolates were recovered from 2400 samples, with the highest prevalence observed in eggs (3.5%), followed by human stool (3.3%), chicken meat (1.8%), and chicken feces (1.1%). Only 35 (11.8%) of the 297 sampled farms were positive for *Salmonella*, and recovery rates differed significantly (*p* = 0.0065) among sample sources. Five serotypes were identified, dominated by *S.* Typhimurium (57.4%), followed by *S.* Enteritidis (14.9%), *S.* Anatum (12.8%), *S.* Stanley (8.5%), and *S.* Agona (6.3%). ERIC-PCR revealed multiple clonal clusters, many containing isolates from mixed sources, indicating circulation of related strains between poultry and humans. All isolates were resistant to ampicillin, with high resistance to tetracycline (76.6%), sulphamethoxazole–trimethoprim (51.1%), and fluoroquinolones. Overall, 80.9% of isolates were multidrug-resistant, with a mean Multiple Antibiotic Resistance Index of 0.29, highest among isolates from chicken feces. Although the prevalence of NTS was low, the presence of genetically related multidrug-resistant strains across the production chain underscores the role of poultry as a reservoir for zoonotic transmission and highlights the need for coordinated One Health surveillance and antimicrobial stewardship strategies in Nigeria.

## 1. Introduction

Non-typhoidal *Salmonella* (NTS) is one of the most important zoonotic pathogens globally and constitutes a major clinical, public health, and antimicrobial resistance (AMR) challenge, particularly in low- and middle-income countries such as Nigeria. NTS causes illness and death in humans and animals and leads to economic losses through healthcare cost, reduced productivity, and food system disruption. Poultry serves as a key reservoir of NTS and plays a central role in human infection through the consumption of contaminated meat, eggs, and poultry-derived products [1,2]. In Nigeria, poultry is widely consumed placing *Salmonella* at the intersection of food safety, disease, and AMR concerns. Globally, *Salmonella* species are responsible for an estimated 93.8 million cases of gastroenteritis and over 150,000 deaths annually [3]. The burden is highest in sub-Saharan Africa, where about 46% of infections are foodborne and NTS is a leading cause of bloodstream infections, accounting for approximately 29.5% of cases and average fatality rates exceeding 20% [4]. In Nigeria, the true burden is likely underestimated because of limited diagnostic capacity, underreporting, and weak surveillance systems, as only a small proportion of infections are laboratory confirmed [5]. Nevertheless, the economic impact remains considerable, with losses attributed to NTS exceeding USD 930 million in 2020 alone [6]. Clinically, NTS infections typically present as self-limiting gastroenteritis in immunocompetent individuals but may progress to invasive non-typhoidal salmonellosis with bacteremia and focal extraintestinal infections in vulnerable populations, including infants, the elderly, and immunocompromised individuals. In sub-Saharan Africa, invasive NTS disease is associated with substantial morbidity and mortality, particularly among children and severely ill adults [7,8,9]. Many infections remain asymptomatic, making detection and control difficult [10] and underscoring the need for effective prevention and control strategies at the animal–human interface.

The pathogenicity of *Salmonella* is mediated by chromosomal and plasmid-encoded determinants that facilitate host cell invasion and systemic dissemination. The conserved *invA* gene, which encodes a protein essential for epithelial cell invasion, is widely used as a molecular marker for confirming *Salmonella* isolates [9]. However, virulence gene carriage alone does not necessarily predict clinical outcome, as pathogenicity is influenced by regulatory mechanisms, host susceptibility, and environmental factors [11]. Serotyping based on O, H, and Vi antigens, as defined by the White–Kauffmann–Le Minor scheme, remains central to epidemiological surveillance, as specific serovars differ in host range, disease severity, and transmission pathways [12,13]. Molecular typing approaches, including enterobacterial repetitive intergenic consensus (ERIC)-PCR, further support outbreak investigation and source attribution by resolving clonal relationships among isolates [14]. To date, more than 2700 *Salmonella* serovars have been identified, with approximately 90% of human infections attributed to *Salmonella enterica* subsp. *enterica*, including invasive NTS lineages [9]. In poultry, *Salmonella enterica* subsp. *enterica* serovars Gallinarum and Pullorum are highly pathogenic, causing persistent infections that compromise flock productivity and pose significant economic risks to the poultry industry. While live vaccines targeting these serovars are available and are used at about 4–6 of birds’ life in Nigeria to prevent outbreaks of poultry salmonellosis, killed vaccines containing *S*. Enteritidis and *S*. Typhimurium are introduced at the 12–18-week pre-lay stage if there is concern about egg contamination and food safety [6]. Beyond virulence, the public health significance of *Salmonella* is increasingly shaped by its capacity to acquire and disseminate antimicrobial resistance determinants. Resistance to critically important antimicrobials classified under the World Health Organization (WHO) AWaRe “Watch” and “Reserve” categories—including fluoroquinolones, extended-spectrum cephalosporins, carbapenems, and colistin—has been reported [15]. In Nigeria, these antimicrobials are frequently misused in poultry production, often at sub-therapeutic doses for prophylaxis and growth promotion, promoting the emergence and spread of multidrug-resistant NTS strains. Such resistance complicates clinical management, increases healthcare costs, and heightens the public health risk associated with zoonotic transmission. Consequently, multidrug-resistant *Salmonella* has been prioritized by the WHO as a high-risk pathogen requiring continuous surveillance, particularly within livestock production systems.

Nigeria’s poultry sector represents a critical One Health setting for *Salmonella* transmission and AMR dissemination. It is the most commercialized component of the livestock industry, with an estimated population exceeding 180 million birds and annual production of 650,000 metric tons of eggs and 290,000 metric tons of poultry meat [12,16]. Poultry products contribute approximately 15% of the country’s total animal protein intake [16]. However, production and processing remain largely informal, characterized by poor biosecurity, inadequate hygiene, and weak regulatory oversight, conditions that favor bacterial persistence and dissemination along production and value chains. Occupationally exposed individuals, including poultry farmers, hatchery workers, and slaughterhouse personnel, face increased infection risk through direct contact with birds, fecal material, feathers, and carcasses, particularly where personal protective equipment and hygiene practices are inadequate. Additionally, the use of untreated poultry litters as organic fertilizer and in aquaculture systems contributes to environmental contamination and broader ecosystem exposure.

Despite extensive molecular characterization of *Salmonella* serovars and AMR profiles in poultry systems across Africa, Europe, Asia, North America, and South America [1,17,18,19,20,21,22,23], surveillance and molecular epidemiological data from Nigeria remain limited and geographically uneven. Existing studies are largely concentrated in the southwestern, northcentral, and northwestern regions [12,24,25,26,27,28,29,30,31,32,33,34,35,36,37], while data from southeastern Nigeria remain scarce. Available studies from this region [38,39,40] have predominantly relied on phenotypic and biochemical identification with limited molecular characterization, which may misestimate true prevalence and fail to resolve serovar diversity, molecular epidemiology, and detailed AMR profiles. This knowledge gap constrains risk assessment and hinders the development of targeted, evidence-based interventions aligned with national and international food safety and AMR containment strategies. This study therefore aimed to determine the prevalence, serovar diversity, genetic relatedness, and antimicrobial resistance profile of *Salmonella* isolates from intensively farmed chickens, chicken meat, retail table eggs, and occupationally exposed poultry handlers in Enugu State, southeastern Nigeria.

## 2. Materials and Methods

### 2.1. Ethical Approval

Ethical clearance for this study was obtained from the Institutional Animal Care and Use Committee of the Faculty of Veterinary Medicine, University of Nigeria, Nsukka (Protocol No. FVM-UNN-IACUC-2016-056), and from the Ethics Committee of the Enugu State Ministry of Health (Protocol No. ENU/AHW/VOL.3/AB:05/12). All procedures involving animals and humans were conducted in accordance with the Declaration of Helsinki [41]. Written and verbally informed consent was obtained from all participating poultry farm workers prior to sample collection.

### 2.2. Study Area

**Figure 1:**
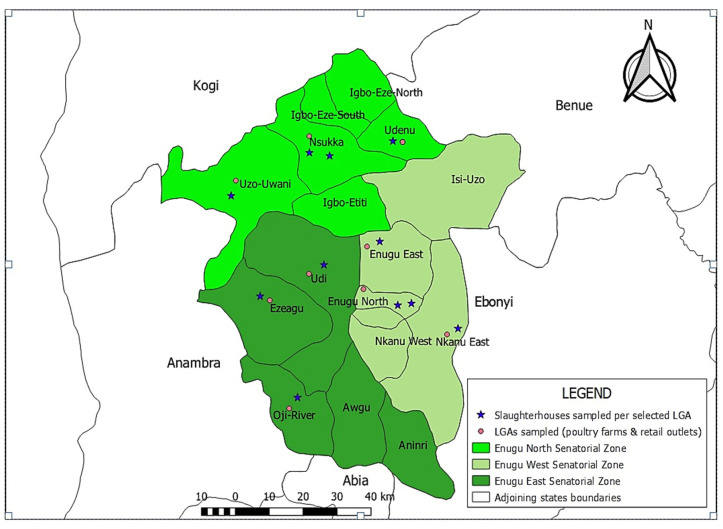
Map of Enugu State southeastern Nigeria showing the Local Government Areas representing 3 senatorial zones (Enugu East, Enugu West, and Enugu North) in which poultry farms, slaughterhouses and egg retail points were sampled.

This study was conducted in Enugu State, southeastern Nigeria (Figure 1), which is situated between latitudes 5°56′–7°55′ N and longitudes 6°53′–7°55′ E. Enugu State is bordered by Ebonyi State to the east, Anambra State to the west, both Abia and Imo States to the south, and both Benue and Kogi States to the north. Administratively, Enugu State comprises three senatorial zones (Enugu East, Enugu West, and Enugu North) and within them, a total of seventeen Local Government Areas (LGAs). Poultry production, predominantly practiced under intensive management systems, continues to reflect a major source of animal protein for the state’s estimated 4 million inhabitants. Each LGA hosts numerous poultry farms and at least one live-bird market where slaughter and processing are frequently performed under suboptimal hygienic conditions.

### 2.3. Study Design and Sampling

This cross-sectional study was conducted over a 12-month period across the three senatorial zones (Enugu East, Enugu West, and Enugu North) of Enugu State to determine the prevalence of *Salmonella* colonization among poultry, poultry products, and poultry farm workers. Sample size was calculated using the formula:

$$N=\frac{Z^{2}\times P(1-P)}{d^{2}}$$

where *Z* = 1.96 at a 5% significance level, *P* = expected prevalence of 26% based on a previous Nigerian study [34], and *d* = desired precision of 5%. The minimum calculated sample size (296 per sample type) was doubled to improve precision. A total of 2400 samples were collected along the poultry production chain, comprising fecal (cecal content)/cloacal swabs (*n* = 1100) from chickens, eggs (*n* = 400), chicken meat (*n* = 600; ~25 g each), and stool samples (*n* = 300) from poultry farm workers. Systematic random sampling was used to reduce bias. Chicken samples were obtained from 297 small- to medium-scale farms (50–2000 birds), representing the predominant production system in the area. Within each farm, birds were selected at fixed intervals (every 20th bird) regardless of health status to capture both symptomatic and asymptomatic carriage. Eggs were collected aseptically from farm storage units and retail outlets at fixed intervals (every 30th egg), including multiple batches to enhance variability. Chicken meat samples were collected from 11 slaughterhouses, where carcasses (chicken breast) were sampled at fixed intervals (every 30th processed bird) during routine operations at different times of the day to account for temporal variation. Poultry farm workers were recruited voluntarily following informed consent, and all consenting individuals present during farm visits were included to capture occupational exposure. Stool samples were self-collected using sterile containers under hygienic guidance and transported in insulated containers.

Sampling sites were distributed across the three senatorial zones of Enugu State (Enugu East, Enugu West, and Enugu North) (Figure 1). Within each zone, multiple Local Government Areas were included, and sampling points were spaced at least 5 km apart to minimize clustering and ensure coverage of diverse poultry production and distribution settings. Overall, the samples represented approximately 15% of the accessible population during the study period. All samples were transported on ice to the Microbiology Laboratory, National Veterinary Research Institute, Vom, Nigeria, and processed immediately or within 24 h of collection.

### 2.4. Isolation and Identification of Salmonella

Isolation of *Salmonella* was performed in accordance with ISO 6579-1:2017 guidelines [42]. All samples were initially pre-enriched in Buffered Peptone Water (BPW) (Oxoid, Hampshire UK). Egg samples were surface disinfected with 70% methanol, after which the contents were aseptically transferred into 20 mL of BPW and homogenized. Approximately 1 g of fecal (cecal content) sample/cloacal swab and 25 g of meat samples were inoculated into 10 mL and 225 mL of BPW, respectively. Pre-enrichment cultures were incubated aerobically at 35 ± 2 °C for 24 h. Selective enrichment was carried out by transferring a loopful of each pre-enrichment culture into Rappaport–Vassiliadis (RV) broth (Oxoid, Hampshire UK), followed by incubation at 42 ± 2 °C for 18–24 h. Thereafter, a loopful from the RV broth was streaked onto Brilliance™ (Oxoid, Hampshire, UK) *Salmonella* Agar (BSA) and Xylose Lysine Deoxycholate (XLD) agar (Oxoid, Hampshire UK), and plates were incubated at 35 ± 2 °C for 24–48 h. Presumptive *Salmonella* colonies—appearing purple on BSA and red with or without black centers on XLD—were subcultured on XLD agar to obtain pure cultures. Purified isolates underwent Gram staining, oxidase test, and biochemical characterization using the Oxoid Microbact™ 12A/12E identification system (Oxoid, Hampshire, UK), following the manufacturer’s instructions. Biochemical tests included lysine and ornithine decarboxylation, citrate utilization, carbohydrate fermentation (glucose, mannitol, and xylose), O-nitrophenyl-β-D-galactopyranoside activity, indole and urease production, acetoin formation, and tryptophan deaminase activity. *Salmonella* Typhimurium ATCC 14028 was used as a positive control, while sterile saline served as a negative control. Presumptive *Salmonella* isolates were stored on nutrient agar slants at 4 °C for no longer than 72 h pending molecular confirmation.

### 2.5. Molecular Confirmation of Salmonella

DNA was extracted from 24 h cultures on tryptone soya agar (Oxoid, Basingstoke, UK) using a heat lysis method [43]. Briefly, two colonies were suspended in 200 µL of molecular-grade water, vortexed, heated at 100 °C for 10 min, and centrifuged at 12,000 rpm for 2 min. The resulting supernatant containing genomic DNA was harvested and quantified using a NanoDrop Lite 1000 spectrophotometer (Thermo Fisher Scientific, Waltham, MA, USA), then stored at −80 °C until further analysis. Conventional polymerase chain reaction (PCR) amplification targeted the *invA* gene, a highly conserved *Salmonella*-specific gene encoding an inner membrane protein essential for epithelial invasion [30]. Each 25 µL PCR consisted of 12.5 µL of 2× DreamTaq PCR Master Mix (Thermo Fisher Scientific, Waltham, MA, USA), 1 µL each of forward and reverse *invA* primers (Inqaba Biotechnical Industries, Pretoria, South Africa) (Table 1), 5.5 µL of nuclease-free water, and 5 µL of template DNA. Primer sequences were adopted from previously validated studies [44] and verified in silico prior to use. Amplification was carried out under the following conditions: initial denaturation at 94 °C for 2 min; 25 cycles of denaturation at 94 °C for 1 min, annealing at 58 °C for 1 min, and extension at 72 °C for 1 min; followed by a final extension at 72 °C for 2 min. Conventional PCR products were resolved on 2% agarose gels prepared in 0.5× TBE buffer, stained with ethidium bromide (1 mg/mL), and visualized under ultraviolet illumination using a UV transilluminator Alliance 4.7 (UVITEC system, (Cambridge, UK). An amplicon size of 284 bp was considered confirmatory for *Salmonella*. *S.* Typhimurium ATCC 14028 and nuclease-free water were included as positive and negative controls, respectively.

### 2.6. Serotyping of Salmonella Isolates

Molecularly confirmed *Salmonella* isolates were serotyped using the Kauffmann–White–Le Minor scheme with polyvalent and monovalent O and H antisera (Statens Serum Institut, Copenhagen, Denmark), in accordance with the manufacturer’s instructions. For serogrouping, bacterial suspensions prepared from 24 h nutrient agar cultures were mixed with polyvalent O antisera (poly A–S + Vi) on clean glass slides. Isolates exhibiting agglutination were further tested with monovalent O antisera (O1, O2, O4, O5, O7, O8, O9, O10, and O12) to determine their specific serogroups. Flagellar (H) antigens were identified using both polyvalent and monovalent H antisera (e.g., H2, H6, HL, and Hgm). Visible granular agglutination occurring within 1 min was recorded as a positive reaction. Serovar designation was based on the combination of O and H antigenic profiles, interpreted according to the Kauffmann–White–Le Minor classification [45]. *S.* Typhimurium ATCC 14028 and sterile saline served as positive and negative controls, respectively.

### 2.7. ERIC-PCR Genotyping of Salmonella Isolates

The genetic relatedness of *Salmonella* isolates was assessed using ERIC-PCR fingerprinting, following the protocol of Fendri et al. [7] with minor modifications. Each 25 µL PCR contained 1 µL each of forward and reverse ERIC primers (20 pmol/L; Inqaba Biotechnical Industries, Pretoria, South Africa) (Table 1), 2.5 µL of 10× PCR buffer, 2.0 µL of 2.5 mmol/L dNTPs, 1.5 µL of 25 mmol/L MgCl_2_, 5 U of Taq DNA polymerase, and 1.5 µL of genomic DNA. Conventional PCR cycling conditions consisted of an initial denaturation at 94 °C for 10 min, followed by 31 cycles of denaturation at 92 °C for 30 s, annealing at 40 °C for 60 s, and extension at 65 °C for 8 min, with a final extension at 65 °C for 8 min. Amplified products (5 µL) were mixed with GelRed™ nucleic acid stain (1:500 dilution) (Biotium Incorporated, Fremont, CA, USA) and resolved on 1.5% agarose gels at 100 V for 1 h. Gels were visualized under ultraviolet light using a Bio-Rad gel imaging system (Bio-Rad, CA, USA). Band sizes were estimated using 1 kb and 100 bp DNA ladders (First Base, Selangor, Malaysia). ERIC-PCR profiles were analyzed using Gel Image System software (Version 4.00) to generate similarity matrices. Cluster analysis was performed using the unweighted pair group method with arithmetic mean (UPGMA) implemented in NTSYS-pc version 2.10 software. Dendrograms were constructed to scale, with isolates considered genetically related if they exhibited ≥95% similarity, corresponding to ≤5% divergence [7].

### 2.8. Antimicrobial Susceptibility Testing

Antimicrobial susceptibility testing of *Salmonella* isolates was performed using the disk diffusion method in accordance with Clinical and Laboratory Standards Institute guidelines for human (CLSI M100-ED35:2025) [46] and animal isolates (CLSI VET01S-ED7:2025) [47]. A total of 14 antimicrobial agents representing six classes were tested. The antimicrobial panel was selected based on WHO AWaRe classification and their relevance in human and veterinary medicine in Nigeria. Tigecycline was included as a last-resort antimicrobial to assess emerging resistance trends of clinical significance. Thirteen antimicrobial agents representing five classes were tested using commercially prepared antibiotic disks (Oxoid, Hampshire, UK). These included β-lactams [ampicillin (10 µg), ceftriaxone (30 µg), cefotaxime (30 µg), meropenem (10 µg), and imipenem (10 µg)], fluoroquinolones [ciprofloxacin (5 µg), ofloxacin (5 µg), and enrofloxacin (5 µg)], phenicols [chloramphenicol (30 µg)], tetracyclines [tetracycline (30 µg), doxycycline (30 µg), and tigecycline (15 µg)], and folate pathway antagonists [sulfamethoxazole–trimethoprim (1.25/23.75 µg)]. Bacterial suspensions were standardized to a 0.5 McFarland turbidity (approximately 1.5 × 10^8^ CFU/mL) and evenly inoculated onto Mueller–Hinton agar plates (Oxoid, Hampshire, UK). Antibiotic disks were applied within 15 min of inoculation, maintaining a minimum distance of 24 mm between disks. While most antimicrobial agents were tested using the disk diffusion method, colistin (polymyxin class) susceptibility was determined using agar dilution due to the known limitations of disk diffusion for polymyxins. Briefly, colistin (Sigma-Aldrich™) was incorporated into Mueller–Hinton agar (Oxoid, Hampshire, UK) at concentrations of 1, 2, and 4 µg/mL, in accordance with CLSI guidelines [46]. Bacterial suspensions were prepared from 3 to 5 well-isolated colonies grown on tryptone soya agar (Oxoid, Hampshire, UK) and adjusted to a 0.5 McFarland turbidity standard (approximately 1.5 × 10^8^ CFU/mL). The suspension was then diluted 1:10 in sterile normal saline. A 10 µL loopful of the diluted suspension was inoculated onto the colistin-supplemented Mueller–Hinton agar plates. All plates were incubated aerobically at 35 ± 2 °C for 16–18 h. *Escherichia coli* ATCC 25922 and *E. coli* ATCC^®^ BAA-3170™ were used as quality control strains for disk diffusion and colistin agar dilution assays, respectively. Interpretation of inhibition zones was performed using CLSI M100-ED35 (2025) [46] for human isolates and CLSI VET01S-ED7 (2025) [47] for animal (chickens, eggs, and meat) isolates, as appropriate. The multiple antimicrobial resistance index (MARI) for each isolate was calculated using the formula a/b, where a represents the number of antimicrobial agents to which the isolate was resistant, and b represents the total number of antimicrobials tested. A value greater than 0.2 indicates a high-risk contamination source where antimicrobials are frequently used [44]. Any isolate resistant to at least one agent in three or more classes of tested antimicrobials was classified as a multidrug-resistant strain [48].

### 2.9. Statistical Analysis

The results of the various tests were entered into Microsoft Excel™ (Microsoft, Redmond, WA, USA). Data on the occurrence frequency of *Salmonella* serotypes and antimicrobial resistance were exported to SPSS version 15.0 (SPSS, Chicago, IL, USA) and GraphPad Prism version 8.3.1 (GraphPad Software, La Jolla, CA, USA) for statistical analysis. Frequencies, percentages, and 95% confidence intervals were calculated as appropriate. Prior to analysis, the assumptions of the chi-square test, including independence of observations and adequacy of expected cell counts, were assessed. Fisher’s exact test was applied where more than 20% of expected frequencies were less than five. Data independence was ensured by including only non-duplicate isolates in all analyses. Associations between categorical variables, including differences in *Salmonella* prevalence across sample types and locations, were evaluated using the chi-square test or Fisher’s exact test, as appropriate. These tests were also used to assess differences in antimicrobial resistance proportions among sample sources, depending on data distribution and expected cell counts. All tests were two-tailed, and statistical significance was set at *p* < 0.05. A post hoc power analysis was conducted to assess the adequacy of the sample size for detecting differences between the expected prevalence (26%) and the observed prevalence (2%) of *Salmonella*. With a total sample size of 2400 and a significance level (α) of 0.05, the statistical power exceeded 99%, indicating a high likelihood of detecting true differences where present.

## 3. Results

### 3.1. Prevalence of Salmonella Serotypes

**Figure 2:**
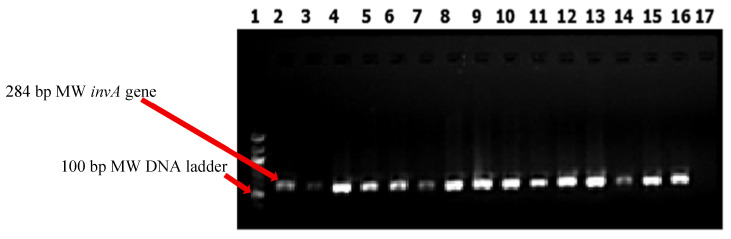
PCR amplification of the *invA* gene in *Salmonella* isolates from poultry production chain and farm workers in Enugu State southeastern Nigeria. Lane 1 contains a 100 base-pair (bp) molecular weight (MW) ladder serving as a size reference. Lane 2 shows amplification of the 284 bp *invA* gene in the positive control strain *Salmonella* Typhimurium ATCC 14028. Lanes 3 to 16 display the amplified 284 bp *invA* gene in the *Salmonella* isolates tested. Lane 17 represents the negative control, where the PCR mix lacked a DNA template.

Out of the 2400 samples collected from poultry birds, poultry products, and farm workers, 47 non-redundant *Salmonella enterica* isolates were recovered and confirmed by detection of the *invA* gene (Figure 2), yielding an overall sample-level prevalence of 2.0% (Table 2). The isolates were obtained from 35 of the 297 farms, corresponding to a farm-level prevalence of 11.8%. Egg samples showed the highest *Salmonella* recovery rate (3.5%), followed by human stool samples (3.3%). Chicken feces and chicken meat yielded recovery rates of 1.1% and 1.8%, respectively. *Salmonella* distribution differed significantly (χ^2^ = 12.36, *p* = 0.0065) among sample sources. The recovered isolates belonged to five *Salmonella* serotypes, with *S.* Typhimurium having the most sample-level prevalence (*n* = 27; 1.12%), followed by *S*. Enteritidis (*n* = 7; 0.3%), *S*. Anatum (*n* = 6; 0.3%), *S*. Stanley (*n* = 4; 0.2%), and *S*. Agona (*n* = 3; 0.1%) (Table 2). The proportional distribution of these serotypes among the 47 isolates was 57.4%, 14.9%, 12.8%, 8.5%, and 6.3%, respectively. A significant difference in occurrence among sample sources was observed only for *S*. Enteritidis (*p* = 0.0078), with eggs recording the highest prevalence. *S*. Typhimurium showed an association with sample source (*p* = 0.051), with eggs and human feces contributing the highest numbers of isolates. No significant (*p* > 0.05) association was observed between *Salmonella* isolation prevalence and the study sites (senatorial zones).

### 3.2. Genetic Relatedness of Salmonella enterica Isolates

**Figure 3:**
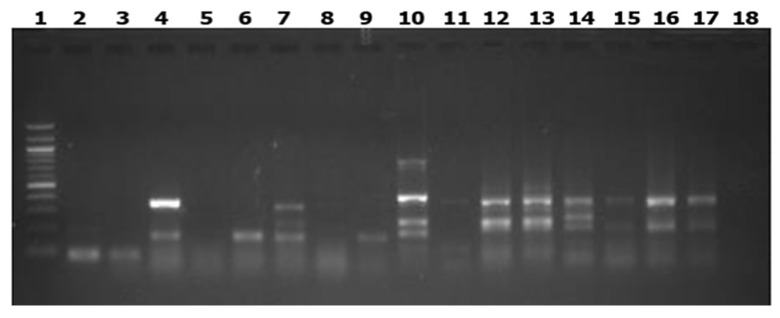
Representative ERIC-PCR fingerprint of different *Salmonella enterica* isolates from poultry production chain and farm workers in Enugu State southeastern Nigeria. Lane 1 contains a 100 bp MW ladder serving as a size reference; Lane 2 shows amplification of the fingerprint in the positive control strain *Salmonella* Typhimurium ATCC 14028. Lanes 3 to 17 display distinct varying number of bands/fingerprints in tested *Salmonella* isolates from different sources. Lane 18 represents negative control, where the PCR mix lacked a DNA template.

**Figure 4:**
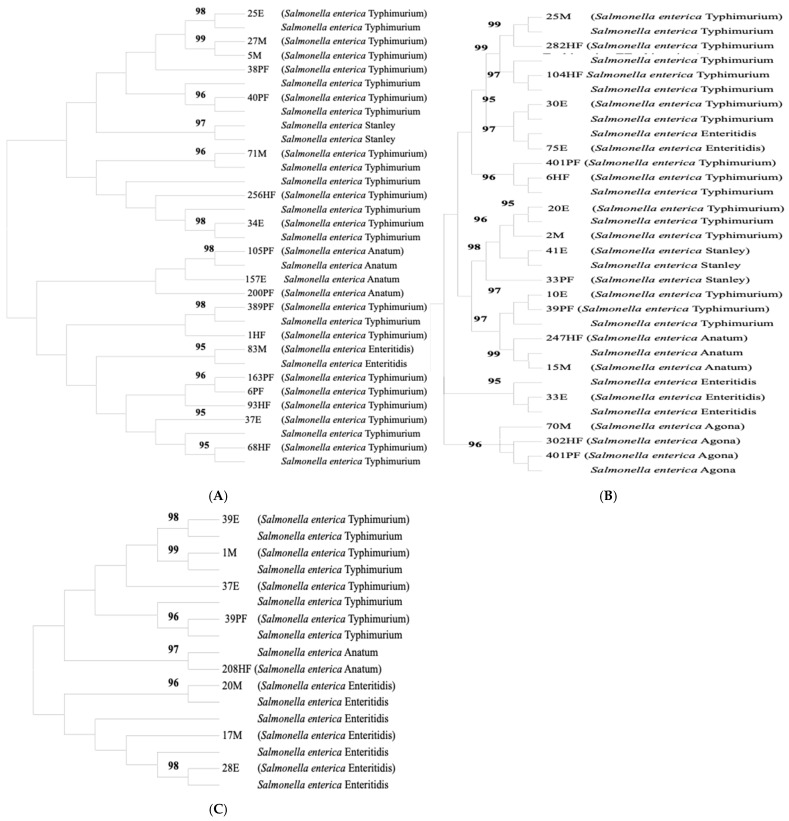
ERIC-PCR dendrogram (spliced into (**A**–**C**)) showing genetic relatedness among *Salmonella* isolates from poultry, poultry products and poultry handlers in Enugu State southeastern Nigeria. Source-ID—E: chicken egg, HF: human feces, PF: poultry feces, M: chicken meat. The numbers on the dendrogram represent similarity coefficients derived from ERIC-PCR profiles using the unweighted pair group method with arithmetic mean (UPGMA), where values closer to 1 indicate higher genetic relatedness among isolates.

ERIC-PCR analysis of the 47 *Salmonella enterica* isolates generated distinct DNA fingerprinting patterns consisting of 1–5 bands, with amplicon sizes ranging from 126 to 980 bp (Figure 3). Cluster analysis based on a similarity coefficient threshold of 0.95 grouped the isolates into 15 major clusters (designated I–XV) and seven singletons (Figure 4A–C and Table 3). Cluster I comprised isolates obtained from egg, meat, and poultry fecal samples, exhibiting a similarity coefficient of 0.99, suggesting close genetic relatedness. Cluster II included strains from meat, human feces, and egg samples (similarity coefficient = 0.96). Cluster III contained *S. enterica* serovar Anatum strains derived from poultry feces and egg samples. Cluster IV consisted of *S. enterica* serovar Typhimurium isolates from poultry feces and human stool, while cluster V contained *S*. Typhimurium isolates from poultry and human fecal samples (163PF, 6PF, and 93HF). Cluster VI grouped *S*. Typhimurium isolates obtained from human feces (68HF) and egg samples (37E), and Cluster VII comprised *S.* Typhimurium isolates from human stool and chicken meat samples. Cluster VIII consisted exclusively of isolates recovered from egg samples. Cluster IX contained *S*. Typhimurium strains from poultry fecal (401PF) and human fecal (6HF) samples, while Cluster X included isolates from egg (20E) and meat (2M) samples. Cluster XI grouped *S. enterica* serovar Stanley isolates from egg (41E) and poultry feces (33PF). Cluster XII was genetically diverse, comprising isolates from egg (10E), poultry feces (39PF), meat (15M), and human fecal (247HF) samples. The seven singletons (designated S1–S7) each contained a single isolate, indicating unique ERIC-PCR profiles (Table 3). Furthermore, Cluster XIII comprised *S. enterica* serovar Agona isolates from meat (70M), human stool (302HF), and poultry feces (401PF). Cluster XIV grouped *S. enterica* serovar Typhimurium isolates from eggs (39E, 37E) and meat (1M), while Cluster XV consisted of *S. enterica* serovar Enteritidis isolates obtained from meat (20M, 17M) and egg (28E) samples.

### 3.3. Antimicrobial Resistance Profile of Salmonella Isolates

Antimicrobial susceptibility testing of the 47 *Salmonella* isolates against 14 antimicrobial agents revealed that resistance to ampicillin was universal (100%) (Table 4). This was followed by resistance to tetracycline (76.6%), sulfamethoxazole–trimethoprim (51.1%), and ofloxacin (48.9%). Lower resistance rates were observed for ciprofloxacin (46.8%), doxycycline (40.4%), and enrofloxacin (34.0%). None of the isolates exhibited resistance to ceftriaxone, cefotaxime, meropenem, imipenem, colistin, tigecycline or chloramphenicol. A statistically significant difference (*p* < 0.05) was observed in resistance to ciprofloxacin, enrofloxacin, and ofloxacin across the four sample sources, with isolates from chicken feces showing the highest resistance levels (Table 4). In contrast, resistance to ampicillin, tetracycline, doxycycline, and sulfamethoxazole–trimethoprim did not differ significantly (*p* > 0.05) among sample sources. No significant (*p* > 0.05) association was detected between *Salmonella* serotypes and antimicrobial resistance profiles. Similarly, resistance to the tested antimicrobial agents did not differ significantly (*p* > 0.05) among the senatorial zones.

All 47 isolates exhibited resistance to at least two antimicrobial agents. Resistance to two, three, four, five, and six antimicrobial agents was observed in 27.7%, 19.1%, 8.5%, 4.3%, and 40.4% of isolates, respectively. Overall, 12 multiresistance patterns (defined as resistance to at least two antimicrobial agents) were identified, of which 10 met the criteria for multidrug resistance (resistance to at least one agent in three or more antimicrobial classes). Among isolates from eggs, three distinct multidrug resistance patterns were observed, with AMP–TET–SXT being the most common (*n* = 6). Isolates from chicken meat and chicken feces exhibited four distinct patterns each, with AMP–TET (*n* = 4) and AMP–ENR–CIP–OFX–TET–DOX (*n* = 5) being the most frequent, respectively. Isolates from human stool showed seven distinct multidrug resistance patterns, with AMP–ENR–CIP–OFX, AMP–OFX–TET–DOX–SXT, and AMP–CIP–OFX–TET–DOX–SXT being the most common (*n* = 2 each).

Overall, 80.9% (38/47) of the isolates were classified as multidrug-resistant (MDR) (Table 5). The MDR prevalence among isolates from eggs, chicken meat, chicken feces, and human stool was 71.4% (10/14), 45.5% (5/11), 66.7% (8/12), and 80.0% (8/10), respectively. The mean Multiple Antibiotic Resistance Index (MARI) across all isolates was 0.29 (range: 0.14–0.43) (Table 5). Isolates from chicken feces exhibited the highest mean MARI value of 0.36 (range: 0.14–0.43), while isolates from eggs, chicken meat, and human stool showed mean MARI values of 0.25, 0.27, and 0.24, respectively.

## 4. Discussion

This study investigated the diversity of *Salmonella* serovars, their genetic relatedness, and antimicrobial resistance patterns along the poultry production chain and among farm workers in Enugu State, Nigeria. The overall *Salmonella* prevalence of 2% indicates low-level circulation among poultry, poultry products, and poultry workers in Enugu State. This estimate is reliable because standard bacteriological (ISO 6579-1:2017) [42] and molecular methods were used. In addition, post hoc analysis indicated that the study (due to high sample size of 2400) had very high statistical power to detect a difference between expected (26%) and observed (2%) *Salmonella* prevalence. Despite the low prevalence, *Salmonella* detection highlights the link between human, animal, and environmental health. These findings are consistent with the One Health framework and highlight the potential for zoonotic transmission along the poultry production chain. The low prevalence may be due to improved hygiene and biosecurity in breeder farms, hatcheries, and poultry farms, which likely reduced non-typhoidal *Salmonella* contamination and occupational exposure. In addition, the apparently healthy status of the sampled birds and the predominance of small-to-medium scale farms (50–2000 birds), where management intensity is relatively low, may have limited pathogen amplification and dissemination [12,22]. Comparably low prevalences have been reported in Nigeria using molecular approaches, including 1.97% in Abia State southeastern region [38], 15.9% in northwestern Nigeria [12], and 8.6% in northcentral Nigeria [30]. In contrast, studies relying solely on phenotypic identification—which may overestimate or underestimate true prevalence—have reported higher and more variable prevalences, ranging from 2% to 27.1% across different regions [25,31,49,50,51,52,53]. Internationally, One Health-oriented studies reported prevalences of 2.0% in Thailand [54], 1.86% in India [55], 14.4% in Ethiopia [22], and 34.3% in Iran [56]. These variations likely reflect differences in sampling strategies, sample types, detection methods, farm management, and biosecurity practices. Variation in *Salmonella* across sample types suggests uneven contamination along the production chain. Although egg contamination was low (3.5%), it remains epidemiologically important because eggs can transmit *Salmonella* during handling or consumption, particularly when undercooked. Egg contamination may occur through vertical transmission, environmental exposure, or handling [57]. Previous Nigerian studies reported egg contamination rates ranging from 1.7% to 33.3% [39,50,58,59]. Lower prevalences reported elsewhere—including 0–3.0% in South Africa, Bangladesh, Chile, United Kingdom, Canada, Japan, Poland, and the USA—have been attributed to improved egg handling and processing practices [60,61,62,63,64,65,66]. In this study, disinfection of eggshells and exclusive culture of egg contents, which possess natural antimicrobial properties, likely contributed to the lower recovery rate [63].

The low prevalence in chicken feces (1.1%) and meat (1.8%) suggest reduced carriage and contamination, possibly due to vaccination and improved farm hygiene. However, meat contamination remains a public health concern because cross-contamination during slaughter and processing is common in many Nigerian facilities. Previous Nigerian studies reported chicken meat contamination rates ranging from 2% to 22.6% [49,67,68], while higher rates have been documented in some countries in Africa, Asia, Europe, North America (Mexico) and South America [69,70,71,72,73,74,75]. Differences likely reflect variation in slaughter hygiene, sample handling, storage conditions, and intensity of cross-contamination. Despite the low fecal carriage rate, *Salmonella* detection in poultry feces remains clinically and environmentally significant. Poultry workers and others are frequently exposed to fecal material, increasing infection risk. Moreover, poultry may act as asymptomatic carriers, intermittently shedding *Salmonella* and contaminating feed, water, and farm environments [25]. The use of uncomposted poultry manure and improper waste disposal may spread *Salmonella* to crops and water bodies, sustaining transmission within the One Health system.

The farm-level prevalence of 11.8% indicates moderate circulation of *Salmonella* within poultry farms in the study area, confirming their role as reservoirs for transmission to humans and the environment. Although lower than the 48%, 47.9% and 43.6% reported by Adetunji et al. [37], Jibril et al. [12] and Fagbmila et al. [25], respectively, this prevalence aligns with reports from other African countries (18.1–50.6%) [17,22,76], South America (26.67%) [19] and Asia (18.0–46.3%) [56,77], but exceeds those from Europe (1.57–8.6%), where control programs are more rigorously implemented [78,79,80]. The moderate farm prevalence observed may be related to small- to medium-scale production systems, where partial intensification and inconsistent biosecurity facilitate bacterial persistence. Detecting *Salmonella* in 3.3% of healthy workers is a public health concern. Asymptomatic carriers may transmit the pathogen to animals, food products, and the environment. Similar carriage rates have been reported in Nigeria [32,34], but *Salmonella* was not recovered from feces of poultry handlers elsewhere [19]. Poor hand hygiene, consumption of contaminated food or water, and close contact with poultry are likely routes of acquisition by farmers in this study.

Five non-typhoidal *Salmonella* serovars (Typhimurium, Enteritidis, Anatum, Stanley, and Agona) were identified, all of which pose public health relevance. This indicates that poultry in the study area serves as a reservoir for diverse pathogenic *Salmonella* strains. This finding is a One Health concern because all detected serotypes cause human foodborne infections [81]. The predominance of *S.* Typhimurium and *S.* Enteritidis is particularly noteworthy, given their global recognition as the leading zoonotic serotypes associated with poultry and poultry products, including in Nigeria [27,34,81,82]. These serotypes have broad range of hosts and comprise both invasive and non-invasive lineages responsible for a substantial proportion of human salmonellosis cases, especially in sub-Saharan Africa, where invasive disease is associated with severe clinical outcomes [1,21,81,83].

Variation in the distribution of *S.* Typhimurium and *S.* Enteritidis suggests heterogeneous transmission within the poultry production system. The higher prevalence of *S.* Enteritidis in eggs, and *S.* Typhimurium in chicken feces and human stool underscore the epidemiological importance of these reservoirs and highlights the critical role of poultry and poultry products in salmonellosis transmission. The predominance of *S*. Enteritidis in eggs is consistent with its well-documented association with egg-related outbreaks and foodborne infections globally [21,81]. This source-specific pattern likely reflects the preferential colonization of reproductive tissues in laying birds, facilitating vertical or trans-shell contamination. Egg-associated *S*. Enteritidis infections are often linked to large outbreaks and may affect vulnerable populations. The detection of *S.* Typhimurium in both poultry and human samples further reinforces its zoonotic potential and suggests that the birds possibly were not vaccinated against these serotypes. It also suggests ongoing bidirectional transmission between animals and humans, consistent with previous One Health studies in Africa and other regions [21,84]. Similar predominance of *S.* Typhimurium and *S.* Enteritidis has been reported in poultry in Nigeria [29,40] and globally [81,85], although regional variations exist. For example, Jibril et al. [12] did not detect these serovars in poultry in northwestern Nigeria, while Raufu et al. [86] reported *S.* Enteritidis, *S.* Eko, and *S.* Hadar as predominant serovars among humans in northeastern Nigeria, underscoring spatial heterogeneity in serotype circulation. The recovery of *S.* Anatum, *S.* Stanley, and *S.* Agona at lower frequencies reflects the increasing diversity of non-typhoidal *Salmonella* circulating in poultry production systems in southeastern Nigeria, in agreement with previous Nigerian and African studies [21,87,88,89]. The lack of significant differences in their occurrence across sample types suggests low-level, sporadic circulation, possibly driven by environmental, feed, or management-related contamination rather than stable host adaptation. The absence of poultry-specific serotypes (*S.* Gallinarum and *S.* Pullorum) may be attributable to routine vaccination against fowl typhoid and pullorum disease in Nigeria [90]. ERIC-PCR showed close genetic relationships among isolates from poultry and humans, with clustering of feces, eggs, meat, and stool samples (≥0.95 similarity), indicating circulation of closely related clones within the production chain. This pattern is likely facilitated by suboptimal biosecurity, cross-contamination during slaughter and processing, and occupational exposure. The recurrent clustering of *S.* Typhimurium from poultry and human sources aligns with its established role as a dominant zoonotic serovar in poultry-associated foodborne transmission [21,81,84]. Similarly, the detection of genetically related *S.* Enteritidis, *S.* Anatum, *S.* Stanley, and *S.* Agona across different matrices supports evidence that poultry and poultry products act as reservoirs of diverse *Salmonella* serovars of public health relevance [1,21,81]. The coexistence of mixed-source clusters and unique singletons suggests both clonal expansion and repeated introduction of distinct strains, potentially through hatcheries, feed, or environmental sources. Nevertheless, clustering patterns were largely serovar-specific, with minimal inter-serovar overlap, and although some clusters comprised isolates from multiple sources, these findings indicate genetic relatedness rather than direct transmission, given the absence of temporal and epidemiological linkage.

Antimicrobial susceptibility profiling revealed universal resistance to ampicillin (100%), with high resistance rates to tetracycline (76.6%) and sulfamethoxazole–trimethoprim (51.1%). Similar patterns have been reported in Nigeria and other low- and middle-income countries where these drugs are widely used in poultry production [34,88]. This resistance reduces the effectiveness of these first-line drugs and increases the risk of treatment failure and prolonged bacterial shedding. Of particular concern is the substantial resistance to fluoroquinolones—ofloxacin, ciprofloxacin, and enrofloxacin (34.0–48.9%)—given their classification in the WHO AWaRe “Watch” group as critically important for human medicine [15]. This resistance likely results from frequent use of these drugs in poultry production as well as in humans in Nigeria, including the study area [91]. The significantly higher fluoroquinolone resistance among isolates from chicken feces suggest heterogeneous on-farm antimicrobial exposure and selection, with resistant strains potentially disseminating along the poultry production chain through carcass processing, handling, and environmental contamination, thereby facilitating human exposure within a One Health context. Although ofloxacin was not commonly used in Nigeria at the time of isolate recovery, cross-resistance is biologically plausible, as the use of one fluoroquinolone can select for resistance across the class, highlighting the interconnectedness of antimicrobial use in animal and human sectors. In contrast, the absence of resistance to third-generation cephalosporins, carbapenems, and colistin may reflect limited use of these last-resort antimicrobials in the sampled farms at the time of isolate recovery. However, this finding should be interpreted cautiously because recent reports indicate inappropriate veterinary use of these antimicrobials and increasing resistance, including plasmid-mediated resistance, among foodborne pathogens in Nigeria, particularly in the southeastern region [92,93]. The observed antimicrobial resistance patterns further suggest the circulation of resistant strains across these interfaces, emphasizing the need for integrated surveillance and coordinated antimicrobial stewardship strategies. The absence of chloramphenicol resistance likely reflects its long-standing ban and reduced use. However, its recent reintroduction in human medicine for the treatment of typhoidal salmonellosis may create new selective pressure and therefore require monitoring. The absence of significant associations between *Salmonella* serotypes or senatorial zones and antimicrobial resistance patterns may be due to the relatively small number of isolates, which limited the statistical power to detect serovar-specific resistance patterns. However, the finding may also indicate that antimicrobial selection pressure, rather than clonal lineage or geographic location, is the main driver of resistance in the poultry production chain in southeastern Nigeria. The high prevalence of multidrug resistance (80.9%), the presence of 12 resistance patterns, and elevated MARI values (0.24–0.36), particularly among chicken fecal isolates (0.36), indicate sustained exposure to multiple antimicrobial classes. This likely reflects the common use of antimicrobial combinations for prophylaxis and treatment in the Nigerian poultry sector. However, information on feed composition and medication records was not collected in this study. Therefore, this explanation should be interpreted cautiously. Despite the low *Salmonella* prevalence, the detection of multidrug-resistant strains has important public health implications. Even low-level circulation in poultry systems may allow these strains to persist and spread over time.

## 5. Conclusions

This study provides baseline data on the prevalence, serovar diversity, genetic relatedness, and antimicrobial resistance of *Salmonella* at the poultry–human interface in Enugu State southeastern Nigeria. Although prevalence was low, the detection of MDR strains underscores the importance of continued One Health-oriented surveillance. Integrated monitoring in poultry systems is essential for early detection and risk control. The potential limitations of this work include that only the internal egg contents were analyzed after surface disinfection; therefore, potential *Salmonella* contamination on eggshell surfaces may have been underestimated. The lack of antimicrobial use data and risk factor (such as biosecurity practices) analysis limits interpretation of resistance drivers. Absence of whole-genome sequencing prevented finer resolution of transmission pathways and resistance determinants. ERIC-PCR should be interpreted cautiously because they cannot confirm direct transmission. Nonetheless, this study delivers robust, region-specific data critical for strengthening AMR surveillance and informing antimicrobial stewardship.

## Figures and Tables

**Table 1:** Primers used in detection of targeted *invA* and ERIC-PCR.

Target Gene	Primers	Amplicon Size (bp)	Reference
*invA*	F: 5′-GTGTTA TCGCCACGTTCGGGCAA-3′	284	[44]
	R: 5′-TCATCGCACCGTCAAAGGAACC-3′		
*ERIC*	F: 5′-ATGTAAGCTCCTGGGGATTCAC-3′	400	[7]
	R: 5′-AAGTAAGTACTGGGGTGAGCG-3′		

*ERIC*: Enterobacteria Repetitive Intergenic Consensus; PCR: polymerase chain reaction.

**Table 2:** Prevalence of *Salmonella* in poultry birds, products and farm workers in Enugu State Nigeria.

Sample	Number	Number (%) of *Salmonella enterica* Serotype					Total (%; 95% CI)
		*S.* Typhimurium	*S.* Enteritidis	*S.* Anatum	*S.* Stanley	*S.* Agona	
Chicken feces	1100	7 (0.6)	0 (0.0)	2 (0.2)	2 (0.2)	1 (0.1)	12 (1.1; 0.48–1.72)
Eggs	400	7 (1.8)	4 (1.0)	1 (0.3)	2 (0.5)	0 (0.0)	14 (3.5; 1.7–5.3)
Chicken Meat	600	6 (1.0)	3 (0.5)	1 (0.2)	0 (0.0)	1 (0.2)	11 (1.8; 0.74–2.86)
Human stool	300	7 (2.3)	0 (0.0)	2 (0.7)	0 (0.0)	1 (0.3)	10 (3.3; 1.28–5.32)
Total	2400	27 (1.12)	7 (0.3)	6 (0.3)	4 (0.2)	3 (0.1)	47 (2.0; 1.44–2.56)

*S*: *Salmonella*; CI: Confidence interval.

**Table 3:** Relationship of *Salmonella* from poultry production chain and farm workers in southeast Nigeria.

Clusters	*Salmonella enterica* Serotype	Strain ID (N = 47)	Source
I	*S.* Typhimurium	25E, 5M, 27M, 38PF	Egg
S1	*S.* Typhimurium	40PF	Chicken feces
S2	*S.* Stanley	134E	Egg
II	*S.* Typhimurium	34E, 71M, 256HF	Egg, Chicken meat, Human stool
III	*S.* Anatum	157E, 105PF, 200PF	Egg, Chicken feces
IV	*S.* Typhimurium	389PF, 1HF	Chicken feces, Human stool
S3	*S.* Enteritidis	83M	Chicken feces
V	*S.* Typhimurium	6PF, 163PF, 93HF	Chicken feces, Human stool
VI	*S.* Typhimurium	37E, 68HF	Egg, Human stool
VII	*S.* Typhimurium	25M, 282HF	Chicken meat, Human stool
S4	*S.* Typhimurium	104HF	Human stool
VIII	*S.* Typhimurium	30E	Egg
	*S.* Enteritidis	75E	Egg
IX	*S.* Typhimurium	401 PF. 6HF	Chicken feces, Human stool
X	*S.* Typhimurium	20E, 2M	Egg, Chicken meat
XI	*S.* Stanley	41E, 33PF	Egg, Chicken feces
XII	*S.* Typhimurium	10E, 39PF	Egg, Chicken feces
	*S.* Anatum	15M, 247HF	Chicken meat, Human stool
S5	*S.* Enteritidis	33E	Egg
XIII	*S.* Agona	70M, 401PF, 302HF	Chicken meat, Chicken feces, Human stool
XIV	*S.* Typhimurium	37E, 39E,1M	Egg, Chicken meat
S6	*S.* Typhimurium	39PF	Chicken feces
S7	*S.* Anatum	208HF	Human stool
XV	*S.* Enteritidis	28E, 17M, 20M	Egg, Chicken meat

*S*: *Salmonella*, ID: identity, I–XV: Cluster 1 to Cluster 15, S1–S7: Singleton 1 to Singleton 7, E: egg, M: chicken meat, PF: poultry feces, HF: human feces. Clusters were defined using ERIC-PCR similarity coefficients ≥95% using unweighted pair group method with arithmetic mean (UPGMA) analysis.

**Table 4:** Antimicrobial resistance profile of *Salmonella* isolates from poultry production chain and farm workers in southeast Nigeria.

Antimicrobial Class	Antimicrobial Agents	Number (%) of Resistant Isolates				Total (N = 47)	*p* Value
		Egg (N = 14)	Meat (N = 11)	Chicken Feces (N = 12)	Human Stool (N = 10)		
β-lactams	Ampicillin	14 (100)	11 (100)	12 (100)	10 (100)	47 (100)	NC
	Ceftriaxone	0 (0)	0 (0)	0 (0)	0 (0)	0 (0)	NC
	Cefotaxime	0 (0)	0 (0)	0 (0)	0 (0)	0 (0)	NC
	Meropenem	0 (0)	0 (0)	0 (0)	0 (0)	0 (0)	NC
	Imipenem	0 (0)	0 (0)	0 (0)	0 (0)	0 (0)	NC
Fluoroquinolones	Ciprofloxacin	4 (28.6)	5 (45.5)	10 (83.3)	3 (30)	22 (46.8)	0.024 *
	Enrofloxacin	4 (28.6)	2 (18.2)	10 (83.3)	0 (0)	16 (34)	0.0002 *
	Ofloxacin	4 (28.6)	5 (45.5)	10 (83.3)	4 (40)	23 (48.9)	0.039 *
Tetracyclines	Tigecycline	0 (0)	0 (0)	0 (0)	0 (0)	0 (0)	NC
	Tetracycline	14 (100)	7 (63.6)	8 (66.7)	7 (70)	36 (76.6)	0.102
	Doxycycline	4 (28.6)	3 (27.3)	5 (41.7)	7 (70)	19 (40.4)	0.155
Phenicols	Chloramphenicol	0 (0)	0 (0)	0 (0)	0 (0)	0 (0)	NC
Polymyxins	Colistin	0 (0)	0 (0)	0 (0)	0 (0)	0 (0)	NC
Folate pathway antagonists	Sulfamethoxazole–Trimethoprim	6 (42.9)	5 (45.5)	5 (41.7)	8 (80)	24 (51.1)	0.232

N: number of isolates; NC: not computable; * signifies significant difference across a row at *p* < 0.05; *p* values represent comparisons of resistance proportions across sample sources using chi-square or Fisher’s exact test, as appropriate.

**Table 5:** Antimicrobial resistance patterns and antimicrobial resistance indices of *Salmonella* isolates.

SN	Antimicrobial Resistance Pattern	No. of Antimicrobials (MARIs)	Eggs (N = 14)	Meat (N = 11)	Chicken Feces (N = 12)	Human Stool (N = 10)	Total (N = 47)	No. of Antimicrobial Class	No. (%) of MDR Strains
1	AMP-TET	2 (0.14)	4	4	0	1	9	2	38 (80.9)
2	AMP-SXT		0	2	2	0	4		
3	AMP-TET-DOX	3 (0.21)	0	0	0	1	1		
4	AMP-TET-SXT		6	0	0	1	7	3	
5	AMP-CIP-SXT		0	0	0	1	1		
6	AMP-ENR-CIP-OFX	4 (0.29)	0	0	2	0	2	2	
7	AMP-OFX-DOX-SXT		0	0	0	2	2	3	
8	AMP-OFX-TET-DOX-SXT	5 (0.38)	0	0	0	2	2	4	
9	AMP-ENR-CIP-OFX-TET-DOX	6 (0.43)	0	2	5	0	7	3	
10	AMP-ENR-CIP-OFX-TET-DOX		4	0	0	0	4		
11	AMP-ENR-CIP-OFX-TET-SXT		0	0	3	0	3	4	
12	AMP-CIP-OFX-TET-DOX-SXT		0	3	0	2	5		

N: total number, MDR: multidrug-resistant, MARIs: multiple antimicrobial resistance indices, N: total number, AMP; ampicillin, TET: tetracycline, SXT: sulfamethoxazole–trimethoprim, DOX: doxycycline, CIP: ciprofloxacin, ENR: enrofloxacin, OFX: ofloxacin.

## Data Availability

The original contributions presented in this study are included in the article. Further inquiries can be directed at the corresponding authors.

## Abbreviations

The following abbreviations are used in this manuscript:

NTS	Non-typhoidal *Salmonella*
WHO	World Health Organization
AWaRe	Access, Watch, Reserve
PCR	Polymerase chain reaction
ERIC	Enterobacterial Repetitive Intergenic Consensus
AMR	Antimicrobial resistance
MDR	Multidrug-resistant
MARI	Multiple antimicrobial resistance index
